# A Narrative Review Comparing Nutritional Screening Tools in Outpatient Management of Cancer Patients

**DOI:** 10.3390/nu16050752

**Published:** 2024-03-06

**Authors:** Delia Gil-Andrés, Luis Cabañas-Alite

**Affiliations:** 1 Internal Medicine Department, Manises’ Hospital, Av. De la Generalitat Valenciana, 50, 46940 Manises, Spain; 2 Faculty of Health Sciences, Miguel de Cervantes European University, C. del Padre Julio Chevalier, 2, 47012 Valladolid, Spain

**Keywords:** nutritional screening, outpatient, malnutrition, outpatient oncology

## Abstract

Malnutrition during cancer has a negative impact on prognosis and quality of life. Therefore, it is important to identify those patients at higher nutritional risk to prevent its development. There are nutritional screening tools, such as MUST and NRS-2002, that focus on the patient on admission to hospital. However, most patients will develop malnutrition in the outpatient or ambulatory setting. This study aims to determine which nutritional screening tool is most effective in assessing nutritional risk in the outpatient oncology patient, highlighting the parameters analysed by these tools. Seventeen articles were reviewed, with the most important variables being tumour location, tumour stage, age, and gender, as well as recent weight loss, dietary intake, and digestive disorders. The Nutriscore, NRS-2002, and MUST tools are considered suitable, but the choice varies depending on these parameters. MNA is suitable for elderly patients, while SNAQ was not considered reliable in this population. In conclusion, MUST, NRS-2002, and Nutriscore are suitable tools, but their choice depends on specific characteristics. There is currently no universal tool for nutritional risk assessment in outpatients.

## 1. Introduction

Cancer is characterised by a rapid proliferation of abnormal cells that can spread, encompassing a group of diseases that share common features, such as their genetic origin, resistance to cell death, and their ability to invade surrounding tissues or metastasise to distant organs [1,2]. In 2020, 19.3 million new cases and 10.0 million deaths were reported globally, with breast, lung, colorectal, prostate, and stomach cancer being the most prevalent, according to data from the International Agency for Research on Cancer (IARC) [3].

Among its consequences are impaired nutritional status, known as disease-related malnutrition (DRE), and cancer cachexia [4,5]. DRE is associated with inflammation and is the preliminary step to cachexia, which represents the most severe state of malnutrition in the cancer patient, characterised by muscle loss (with or without loss of fat mass) that cannot be fully reversed with conventional nutritional support, leading to progressive functional deterioration. It is present in up to 80% of patients and is associated with 20% of cancer mortality [6].

Cachexia is caused by an imbalance between protein and energy intake due to reduced appetite, but also by the effects of inflammation and metabolic alterations induced by the tumour itself [2,7]. Loss of muscle mass has been associated with increased dependency, decreased quality of life, and poorer mental health [7,8]. Furthermore, cachexia increases the risk of infectious complications and limits therapeutic options, as these patients often have poorer tolerance to antineoplastic treatments and lack of response [8].

The aim of any nutritional intervention is therefore to avoid this complication. Screening and early detection tools, which quantify the risk of developing malnutrition (nutritional risk) or are useful in its diagnosis, are available to prevent it. These tools use variables that include the measurement of parameters such as body mass index (BMI), percentage of unintentional weight loss (%WL) [9], or dietary intake [10], among others.

The most developed are nutritional screening tools, which seek to identify those patients who may be at increased nutritional risk and require detailed nutritional assessment [11]. In the hospital and ambulatory field, more than 70 NCs exist. However, there is no international consensus on the best tool to identify malnutrition in specific populations, including oncology outpatients [12]. Among the most relevant tools are the Malnutrition Universal Screening Tool (MUST), Nutritional Risk Screening 2002 (NRS-2002), the Malnutrition Screening Tool (MST), Mini-Nutritional Assessment (MNA), Nutritional Control (CONUT), the Short Nutritional Assessment Questionnaire (SNAQ), and Nutriscore, the latter being the only one specifically designed for oncology patients [13].

Once patients at nutritional risk have been identified, an assessment is performed. Currently, this is conducted following the framework established by the Global Leadership Initiative on Malnutrition (GLIM) [11,12,14]. In addition, a second diagnostic method, the Patient-Generated Subjective Global Assessment (PG-SGA), is considered a secondary reference criterion for identifying malnutrition in oncology patients, according to the GLIM criteria [14,15,16,17].

This study aims to determine which of the current nutritional screening tools is most suitable for assessing nutritional risk in ambulatory oncology patients, according to the factors used for assessment or indicated as predictors of DRE.

## 2. Materials and Methods

### 2.1. Literature Research

This study is based on a literature review of information published between 2010 and 2023. This review was carried out according to the PRISMA 2020 statement. The literature search was conducted in the following databases: PubMed, Scopus, Embase, and SciELO, in which a total of 102 articles were found. The search terms used were determined via the PICO method, using the terms “Nutrition Assessment”, “Nutritional Index”, “Mini Nutritional Assessment”, “subjective global assessment”, “malnutrition universal screening tool”, “NRS-2002”, “SNAQ”, “CONUT”, “nutritional screening tools”, “Glim Criteria”, and “Cancer outpatients” in the final route.

### 2.2. Study Eligibility, Selection and Data Extraction

On the one hand, research articles were selected taking into account the following inclusion criteria: (1) original articles, (2) articles published between 2010 and 2023, (3) those written in English, Spanish, or German, (4) articles whose main objective was to evaluate nutritional screening tools, (5) studies conducted among cancer outpatients. On the other hand, exclusion criteria were as follows: (1) studies that did not specifically address the main objective of the review, (2) articles focusing exclusively on hospitalised patients, (3) those repeated in another database and already included in the review, and (4) systematic reviews, narratives, and meta-analyses. 

The first phase of study selection was performed by reviewing titles and abstracts to determine eligibility. Studies that did not meet the inclusion and exclusion criteria at this stage were discarded. Of the remaining studies, a second screening process was carried out on which full articles were reviewed. This process was conducted independently and blindly by two reviewers, who had no access to each other’s decisions, or to reviewer, reference, or author information.

## 3. Results

**Figure 1:**
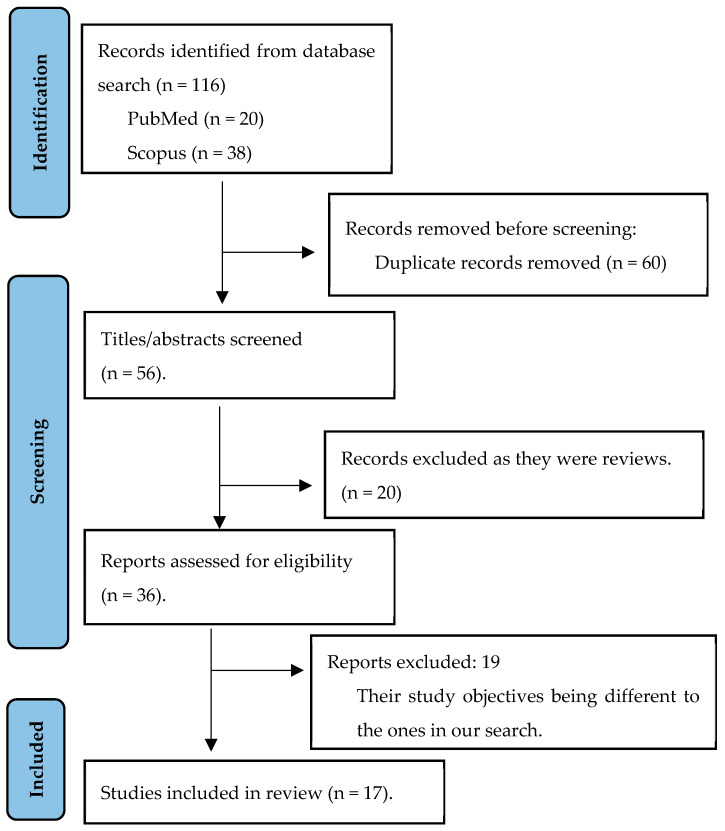
Study data collection: authors’ own creation.

After the literature search, 116 articles were identified. After excluding 99 of them, 17 articles that met the inclusion criteria were retained and included in the final review. The screening process can be seen in Figure 1, in a flow diagram of the search process, adapted from PRISMA. 

As shown in Figure 1, 60 articles were identified as duplicates and were therefore not considered in the final selection. After the second screening stage in which titles and abstracts were analysed, 20 articles were excluded. Consequently, 36 references were pre-selected and underwent a second screening process, which involved a detailed analysis of their relevance to the review. Nineteen references were excluded because, despite addressing the nutritional status of outpatient cancer patients, they had different study objectives to those initially proposed. Of these articles, one was discarded because it was a review. Thus, a total of 17 articles selected for the review met the inclusion criteria and are detailed in Table 1.

### 3.1. Summary of the Studies Included

#### Prevalence of Malnutrition in the Outpatient Cancer Population

Of the final included articles, eleven addressed the prevalence of undernutrition in the outpatient cancer patient [18,19,21,23,24,27,28,29,30,32,34], while five studies additionally focused on assessing nutritional risk [19,20,26,28,29]. Notably, three of these studies considered both prevalence and nutritional risk, thus allowing comparison of both percentages [19,28,29].

In addition, eight of the studies investigated patients affected by malignancies with a higher incidence of malnutrition, such as tumours of the head and neck, upper and lower digestive tract, and lung [18,19,23,24,25,26,30,34]. In addition, two of them focused on patients with metastatic cancer, whose prevalence of malnutrition often increases [22,25]. In contrast, five studies did not differentiate according to tumour type, but included the entire cancer population without discerning tumour type [20,21,27,31,33]. Of these, one focused specifically on the population over 70 years of age [29].

Also, three studies explored other cancer types in addition to digestive, respiratory, and head and neck tumours, such as gynaecological, breast, prostate, and haemostatic cancers [23,28,34].

### 3.2. Comparison with Different Screening Tools

Out of the articles reviewed, five of the studies carried out comparisons between various screening tools and the newly established GLIM criteria used as the reference diagnostic method [18,19,29,30,32]. On the other hand, in seven of the articles, the PG-SGA diagnostic method was applied to assess the nutritional status of patients [21,23,24,30,31,32,34], and in four of them, the PG-SGA method was used to assess the validity of the tools and their suitability in this specific population [23,24,31,34]. In addition, two studies focused on examining the validity of the two versions of this diagnostic method in this specific population, compared with the new GLIM criteria [30,33]. Only one study compared the screening tools with the ESPEN criteria [28]. Another study compared two specific tools, NRS-2002 and MUST, without diagnostic methods and looked at the results of each [27].

### 3.3. Parameters Involved in the Development of Malnutrition

Out of the included articles, seven studies examined the importance of the weight loss parameter as a predictor of undernutrition, highlighting the need to integrate it into screening tools [19,20,22,25,26,27]. In the same context, three of these studies also focused on the importance of reduced intake in nutritional status [19,25,26]. On the other hand, five articles addressed the relevance of body mass index (BMI) in the assessment of nutritional risk [19,20,23,28,31]. In addition, two studies examined how age and sex variables may be associated with nutritional impairment [19,20]. Finally, four studies explored how the nature of the disease, including tumour typology and tumour stage, may contribute to a greater development of malnutrition [21,25,27,28].

## 4. Discussion

Early detection of nutritional risk or DRE is of vital importance to improve the clinical outcomes and quality of life of these patients. It is necessary to know which tool is best suited to the pathology to detect greater risk and thus avoid DRE. These tools should be used from diagnosis and not only for hospital admissions. For this reason, the objective of this research is to assess which of the current tools are most effective in identifying nutritional risk or the prevalence of DRE in outpatients, as well as which factors are considered most accurate for using one NS tool or another. The studies analysed highlight several influential factors in the prevalence and severity of DRE, including characteristics such as tumour type, location, and stage, as well as gender and age, which will be examined below.

### 4.1. Malnutrition Prevalence

In relation to the prevalence of DRE, significant figures stand out that range between 40% and 50%, according to the studies by Sobrini et al. (2021) [29], Tob-berup and Thoresen (2020) [32], Auma et al. (2022) [24], Hettiarachchi et al. (2018) [23], and Gascón et al. (2022) [18]. Likewise, a considerable nutritional risk, around 30%, was identified according to studies that analysed the risk of malnutrition such as those by Kaduka et al. (2017) [34], Hauner et al. (2020) [27], and Bozzetti et al. (2012) [26]. These findings show a clear discrepancy between the values obtained through the use of nutritional screening tools and the diagnostic criteria.

Regarding the criteria for diagnosing malnutrition in outpatient cancer patients, despite the consensus among experts on the GLIM criteria, there is a lack of comparative studies that evaluate the validity of their use in screening tools. Five publications were found that used the GLIM criteria [18,19,29,30,32]. It is noteworthy that in the study by Gascón et al. (2022) [19], a general prevalence of malnutrition of 53.3% was revealed, with significant differences between types of cancer. Most articles used the PG-SGA as diagnostic criteria or as a reference to compare other screening tools [21,23,24,30,31,32,34]. There have been similar studies, such as the NUPAC carried out in Spain in 2005, which showed that 52% of patients had severe malnutrition or were at risk of suffering from it [35]; however, Rossi et al. [36] point out that malnutrition can affect up to 75% of patients, associating variability with the influence of factors related to the disease, such as the type of tumour, stage, and treatment, as well as with demographic (age) and social aspects.

It should be indicated that malnutrition has not always been assessed in the most relevant way. Several screening tools use parameters such as BMI [17,23,34,35] and this is a limitation that should be considered in the future, using much more precise tools to assess nutritional status, such as body composition measured via BIA, muscle function, and physical performance [37,38] or GLIM criteria, effective tools for nutrition assessment and survival prediction in older cancer patients [39]. Other authors have included the assessment of body composition or sarcopenia parameters by computed tomography or DEXA [36,38], but these tools are more time-consuming and costly to implement. In fact, the use of anthropometric measures may provide information on alterations in body composition, especially the reduction of muscle mass, but this is not the case with the use of measures such as weight loss or BMI in cancer patients [36].

Novel approaches to the diagnosis of low muscle mass, muscle function, or sarcopenia have been evaluated for use in nutritional screening tools, with the adoption of criteria that include the assessment of body composition, such as BIA.

### 4.2. Tumour Type

The type and location of the tumour are relevant factors that can affect the development of malnutrition in cancer patients. Several studies have examined the prevalence and risk of malnutrition depending on the type of tumour. For example, Gascón et al. (2022) [19] identified that head and neck tumours, as well as gastrointestinal cancer, have a high incidence of malnutrition (57% and 59% respectively, according to GLIM criteria). Furthermore, other research carried out by Jendretzki et al. (2021) [20] and Hauner et al. (2020) [27] indicates that patients with digestive tumours have the highest risk of malnutrition. In particular, it has been observed that cancers of the stomach, oesophagus, and pancreas show more significant weight loss, with the latter being associated with the greatest deterioration in nutritional status. Furthermore, the study by Hauner et al. (2020) [27] indicates that the specific location of the tumour in the digestive system also has an influence, with tumours located in the upper part of the digestive tract, such as the oesophagus, being more prone to malnutrition than those located in lower parts. In summary, it could be indicated that there are oncological pathologies that relate to weight loss and, therefore, malnutrition. Specifically, it could be said that digestive or pancreatic tumours bring a high risk of weight loss and malnutrition, while other pathologies such as breast cancer do not have this direct relationship with the pathological diagnosis, the risk being associated with the timing of subsequent treatment.

These data are consistent with what has been published by other authors. The NUPAC study revealed that tumours of the oesophagus, stomach, and head and neck are associated with the highest prevalence of weight loss, registering 57%, 50%, and 47%, respectively [35]. Furthermore, the review by Martinovic et al. [40] specifies that patients with head and neck cancer show a prevalence of weight loss that ranges between 25 and 65%, which can increase up to 80% due to various causes such as the interruption of intake due to surgical procedures, as well as the adverse effects of radiotherapy and chemotherapy treatments, such as mucositis, dysphagia, and xerostomia [36,40]. In other studies, the type of malignant disease was not significant for assessing undernutrition using a standardised questionnaire, such as the MNA, because the patients predominantly had diseases such as breast cancer or lymphoma (*n* = 50), compared with small sample sizes for lung cancer (*n* = 6) and head and neck cancer (*n* = 2) [41].

In addition, the risk factors influencing the occurrence of malnutrition in cancer, and more specifically in some types of cancer, are known. In their review, Bossi et al. [36] indicate that this situation is the result of tumour-induced activation of inflammatory pathways, such as TNF-α influence. Other studies report that inflammatory cytokines (i.e., L-1 or IL6) have been proposed as important factors in the development of cancer-associated malnutrition and their variability in different types of tumours, being much more persistent in tumours of the upper digestive tract and less present in tumours such as melanoma or breast tumours [42]. Specific tumours, such as lung and pancreas, present distinct gene expression profiles of inflammatory-inducing factors that may explain why patients with these types of cancer are more susceptible to developing malnutrition, and also asthenia or cachexia [43]. The presence of these pro-inflammatory factors and the symptoms associated with each type of tumour, such as mucositis, dysphagia, or xerostomia, explain the different prevalence of malnutrition in different diseases and the different nutritional risk.

The above data could indicate that the location of the tumour has an impact on the nutritional status of the patient, so the nutritional assessment should discern according to the type of cancer, because some haematological neoplasms have a very significant relationship with malnutrition, such as lung, pancreatic, or upper digestive tract cancer, while others, such as breast cancer, have a less significant relationship with malnutrition.

### 4.3. Age

In the study by Jendretzki et al. (2021) [20], it was observed that young patients were less likely to experience significant weight loss compared with those over 65 years of age. In contrast, the study by Kaduka et al. (2017) [34] identified age as a predictor of malnutrition and cachexia. These findings suggest that although age is not directly related to weight loss in general, it may influence the risk of significant weight loss in patients. Advanced age is identified as a significant risk factor for the development of malnutrition [44], with an average prevalence of 44.6% in elderly patients [36].

### 4.4. Gender

Women experience greater weight loss, as revealed in the study by Jendretzki et al. (2021) [20]. This is possibly associated with nutritional difficulties related to treatment. This is linked to the disparity in the incidence of different types of cancer between both sexes, with breast and uterine cancer being more prevalent in women, while prostate, oesophageal, and colorectal cancer are more prevalent in men, according to Opanga et al. (2017) [21]. The prevalence of malnutrition varies significantly between the sexes due to the greater propensity for malnutrition with cancers of the gastrointestinal system compared with those of the reproductive system. As a consequence, among patients, it was observed that a higher percentage of women were well nourished, while 54.7% of men presented severe malnutrition [21]. The relationship between gender and prevalence of DRE seems to be unclear, being mainly influenced by the patient’s type of tumour.

### 4.5. Tumour Stage

Tumour stage has a significant influence on the nutritional status of cancer patients, according to the study by Opanga et al. (2017) [21]. It was observed that the majority of patients in advanced stages were malnourished, in contrast to those in early stages. This also influences the effectiveness of nutritional treatment. In advanced stages, nutritional support is less effective, and the efficacy of pharmacological treatment plays a more crucial role, according to Bozzetti et al. (2017) [25]. In contrast, in the early stages, dietary intervention is more significant.

Furthermore, Bozzetti et al. (2012) [26] observed that factors other than the nature of the disease and the tumour stage could predict a high degree of malnutrition, according to the NRS-2002 nutritional assessment system.

The reasons why this may occur are varied, but specifically it may stem from an increase in the pro-inflammatory factors and cytokines listed above, the levels of which increase with tumour size [42,45]. In addition, in advanced stages, obstruction (i.e., head and neck or bowel cancer), functionality changes (i.e., asthenia or cachexia), and severe symptoms (i.e., loss of appetite or steatorrhoea in pancreatic cancer) are more common tumour-related mechanisms of malnutrition than are found in early stages [21,26,45].

### 4.6. Nutritional Status Assessment Factors in Nutritional Screening Tools

It seems relevant to consider decreased intake and recent weight loss (in the last 3–6 months). In this line, Jedretzki et al. (2021) [20] revealed an adequate correlation between results obtained using the MUST tool and variable weight loss, evidencing a weight loss of 44%, while 36% of patients showed malnutrition. However, the patient’s body mass index (BMI), although widely used as a universal parameter, is not an accurate indicator of malnutrition. Hettiaracchi et al. (2018) [23] also noted that BMI misclassified 30 patients, but this error decreased when considering weight loss and performing a full nutritional screening using the MUST tool, misclassifying only 9 patients. Despite its limitations, according to the study by Kaduka et al. (2017) [34], BMI could identify more patients at risk of malnutrition compared with the old ESPEN criteria. Consequently, they concluded that it is important to continuously review the different nutritional screening tools.

However, as stated by Bozzetti et al. (2017) [25], both weight loss and decreased food intake could be key parameters for assessing cancer patients’ nutritional status, along with other clinical variables such as anorexia and digestive disorders. As early as 1980, Dewys et al. (1980) [46] noted that an involuntary reduction of more than 5% of total weight was associated with a decrease in survival, and this weight loss could be used as a prognostic value. In recent years, this factor has been widely used, ranging from 42.25% weight loss associated with cancer-induced anorexia [35] to 74% weight loss between 10–20% of initial weight [47]. Conversely, the review by Bullock et al. (2021) suggested an association between low BMI and unfavourable clinical outcomes, especially in lung cancer patients [48]. It could be suggested that BMI remains the only parameter used to determine nutritional status, despite its acknowledged limitations in adequately identifying malnutrition in these patients.

### 4.7. Choice of Nutritional Screening Tool

After evaluating the variables analysed with the different NS tools to discern which parameters best predict DRE, we analysed which NS tools would best determine nutritional risk. Studies such as that by Gascón et al. (2022) [19] indicate that Nutriscore is useful in the oncology population, especially in head and neck cancer patients. However, it turns out to be less effective in identifying malnutrition in cases of colorectal cancer. In comparison, Hauner et al. (2020) [27] evaluated MUST and NRS-2002 in outpatient cancer patients, observing that both were adequate, although their effectiveness varied depending on the type of tumour. It was observed that patients with digestive tumours had a higher prevalence of malnutrition according to MUST, reaching 46.6%, while NRS-2002 determined a higher percentage of 63.3% malnutrition in patients with hematopoietic tumours. It seems important to consider in this context that the type of tumour has also an impact on the selection of a more appropriate nutritional screening tool according to its features.

Neither tool, MUST nor NRS-2002, was been designed specifically for oncology patients, which implies in their formulation a lack of a direct approach to aspects related to the nature of the tumour. Despite this, both tools consider essential dietary parameters in the prediction of malnutrition, as previously described, such as weight loss in the case of MUST and decreased intake in the case of NRS-2002, including other relevant factors. Notwithstanding, Nutriscore is designed specifically for cancer patients, incorporating aspects of the nature of the tumour, such as its type and the cancer treatment. In addition, it addresses essential dietary parameters such as weight loss and reduced intake, which are also considered by NRS-2002. However, these tools may be limited as they do not take into account the person’s functionality and age or other aspects of the disease such as tumour stage or disease status.

The consideration of age adds relevance to the choice of specific tools, such as the MNA, which was shown by Sobrini et al. (2021) [29] to be effective in older cancer patients. Despite not being designed exclusively for this population and not addressing specific aspects of oncological pathology, the MNA tool includes essential parameters previously described, such as anorexia, recent weight loss, and patient functionality, and performs a detailed analysis of daily dietary intake, among other aspects.

Furthermore, it is necessary to highlight that the MUST screening tool has been widely used to assess the risk of malnutrition, determining its validity for oncology outpatients. In this regard, multiple authors such as Gascón et al. (2022) [19], Kaduka et al. (2017) [28], Hauner et al. (2020) [27], Hettiarachchi et al. (2018) [23], Jendretzki et al. (2022) [20], and Auma et al. (2022) [24] agree that MUST is a suitable tool for determining undernutrition risk, preferring it over the PG-SGA method due to its ease of application and not requiring specialised training. Although Auma et al. (2022) [24] do not make a clear distinction between PG-SGA and MUST, they emphasise the importance of their implementation in both diagnosis and follow-up, rather than the choice between them.

In the case of the SNAQ questionnaire, Helfenstein et al. (2016) [22] concluded that it is not an adequate predictive tool to identify nutritional risk in ambulatory cancer patients, as it does not take into account specific aspects such as tumour stage and adverse treatment effects.

According to the review by Serón-Arbeloa et al. (2022) [49], given the diversity of the disease, there is no single screening tool that can predict clinical outcomes in all patient groups and care settings. This review also compared different tools specifically addressed to the oncology population, predominantly the use of SGA and PG-SGA, with MNA-SF, MST, MUST, and NRS-2002 having been recognised as being useful. In another analysis focused on elderly oncology patients, it was observed that the most widely used nutritional assessment tool in this specific population was the MNA [50]. However, in the review by Bullock et al. (2022), which contrasts several tools, the authors did not identify a single tool suitable for detecting the risk of malnutrition in adult oncological patients [48].

This review stands out for its ability to gather information on an understudied topic, thus opening doors to future research on this subject and highlighting the need for more scientific literature on this pathological entity which is prevalent among oncology patients. Similarly, this review helps to determine the best available evidence on currently validated screening tools. It also provides guidance for health professionals involved in the patient care process. However, there are some methodological limitations that need to be addressed. The time available to carry out this review was limited, which affected the length of the literature search; it ran from May to December 2023, including articles in English, Spanish, and German, thus incurring a potential selection bias that could have had an impact on the results of this research.

Finally, this area has not been sufficiently studied, so the literature does not yield solid conclusions and will need to be supported by further studies. Future nutritional screening tools and studies should consider in their scoring systems other diseases that involve nutritional risk or nutritional complications, such as cachexia or sarcopenia, and patients with chronic disease (diabetes mellitus, hypertension), weight-control medication use, or inflammatory digestive diseases. These conditions and their influence on malnutrition in outpatients could not be assessed in this review because the data from the studies were insufficient or associated comorbidities were not assessed as part of the nutritional screening tools.

## 5. Conclusions

Malnutrition in these patients is influenced by various parameters such as tumour type and location, cancer stage, and age, as well as dietetic and dietary aspects such as recent weight loss, dietary intake, and digestive disorders. The gender factor does not seem to play a role in patient malnutrition.

Therefore, the choice of nutritional screening tool for oncological outpatients should consider these factors. It is essential to adjust the choice to the particularities of the disease and the patient in order to obtain accurate results. Among the tools evaluated, Nutriscore is effective in identifying nutritional risk in patients with head and neck tumours, but not in cases of colorectal cancer. Although it addresses essential aspects, it does not consider other important aspects for assessing nutritional status. The same applies to MUST and NRS-2002, which are useful, although their efficacy may vary according to the type of tumour. Furthermore, MNA shows good results in elderly cancer patients, while SNAQ is not reliable for predicting malnutrition and is discarded as a baseline.

More research is needed on nutritional screening tools for ambulatory cancer patients, highlighting the urgent need to carry out more studies to evaluate and compare tools, as well as to develop new ones that are better adapted to this pathology, using the factors indicated in this study.

## Figures and Tables

**Table 1:** Summary of studies included in the review.

Autor (Year)	*n*	Design	Objective Intervention	Intervention	Time	Conclusions
Gascón et al. (2022) [18]	165	Cross-sectional	Purchase of different screening tools following GLIM criteria.	Head and neck, upper digestive tract, and colorectal tumours.	8 months	Nutriscore and MNA-SF showed better performance in patients with high-risk tumours and older people. MUST showed high specificity and sensitivity in the detection of malnutrition.
Gascón et al. (2022) [19]	165	Longitudinal	To assess the ability of GLIM criteria to predict mortality in outpatients.	Head and neck, upper digestive tract, and colorectal tumours.	6 months	GLIM criteria identify malnutrition in more than half of oncology outpatients and are useful in predicting clinical outcome: increased treatment toxicity, increased mortality, and tumour progression.
Jendretzki et al. (2022) [20]	311	Cross-sectional	To identify nutritional risk in groups of patients in an outpatient center.		8 months	Establishes the MUST tool as a valid screening method. Unintentional weight loss is highlighted as a relevant parameter, especially in overweight patients.
Opanga et al. (2017) [21]	471	Cross-sectional	To assess the nutritional status of patients in Kenya on active treatment using PG-SGA.	Outpatients.		PG-SGA is recommended for its efficacy in detecting malnutrition. The prevalence of malnutrition varies according to tumour type, sex, and tumour stage, being higher in advanced stages.
Helfenstein et al. (2016) [22]	118	Longitudinal	To assess the validity of the SNAQ tool in detecting impending weight loss.	Patients with metastatic cancer.	3 months	SNAQ is not a suitable tool for predicting nutritional status evolution.
Hettiarachchi et al. (2018) [23]	100	Cross-sectional	To determine the prevalence of malnutrition and compare different screening methods with the PG-SGA tool.	Breast, ovarian, prostate, lung, gastrointestinal, and leukaemia tumours.		Prevalence of malnutrition in 45% of patients. The use of MUST was validated, presenting a sensitivity of 89.7% and a specificity of 98.2%, with a positive predictive value of 92.9% and a negative predictive value of 89.7%. Its implementation in clinical practice is recommended as it is simple and does not require training, unlike PG-SGA. BMI was disregarded because of its limited ability to detect malnutrition, misclassifying 30 patients. However, %PP demonstrated adequate specificity and positive predictive value.
Auma et al. (2022) [24]	188	Cross-sectional multicentred	To evaluate the efficacy of MUST and PG-SGA in detecting nutritional risk to improve early identification and management of malnutrition.	Patients with head and neck, respiratory, and gastrointestinal cancers.		MUST and PG-SGA are suitable for the detection of nutritional risk. The results revealed a sensitivity of 83.1% and a specificity of 85.7% for MUST. However, it underestimated nutritional risk in patients with oedema or ascites. The PG-SGA demonstrated a sensitivity of 92.4% and a specificity of 72.5%, being highly sensitive, but its accuracy is influenced by the experience of the observer, which may affect its reliability in nutritional assessment.
Bozzetti et al. (2017) [25]	1000	Longitudinal	Longitudinal assessment of nutritional status using the NRS-2002 tool to screen for the presence of nutritional risk in outpatients.	Solid tumours of oesophagus, stomach, pancreas, small intestine, colon, lung, and head–neck at any stage.	3 years	Gastrointestinal tumours, such as oesophageal, stomach, and pancreatic tumours, caused greater weight loss, with patients affected by oesophageal and pancreatic cancer being at higher nutritional risk. The severity of their weight loss was found to be related to anorexia. Both weight loss and decreased dietary intake, are relevant parameters in the assessment of nutritional status. In addition, weight loss was related to the type of tumour, the stage of tumours, and the ECOG scale. Although nutritional support is less effective in advanced stages, initiating it in early stages is essential for these patients
Bozzetti et al. (2012) [26]	1453	Longitudinal multicentred	To identify nutritional risk and determine patterns of nutritional risk scores in outpatients.	NRS-2002 in patients with solid tumours of the oesophagus, stomach, pancreas, small bowel, colon, lung and head–neck.	4 years	NRS-2002 is an effective tool to detect malnutrition, showing a prevalence of 32%. Tumour location, performance on the ECOG scale, and symptomatology are factors that predict a high score on the NRS-2002. In addition, clinical variables such as weight loss, anorexia, ECOG, and gastrointestinal disorders are indicative of nutritional risk.
Hauner et al. (2020) [27]	765	Longitudinal multicentred	To assess the frequency of undernutrition in outpatients.	NRS-2002 MUST	11 months	Both NRS-2002 and MUST are useful tools, although their efficacy may vary according to tumour type and MUST shows lower sensitivity and specificity than NRS-2002. The prevalence of malnutrition varies according to the type of tumour was 34.9% according to MUST, with a higher incidence in digestive tumours, while the NRS-2002 presented a prevalence of 29.1%, highlighting the relevance in haematopoietic tumours. These discrepancies in the data are due to differences in classification and scoring criteria.
Kaduka et al. (2017) [28]	512	Cross-sectional multicentred	To study the risk of malnutrition and factors associated with malnutrition and cachexia to inform cancer management in Kenya.	Breast, cervical and digestive tract cancer at various stages, especially advanced stages.	1 month	Malnutrition affects 33.1% of patients, revealing that tumour location and treatment affect the prevalence of cancer-related malnutrition. The MUST screening tool is proposed for early identification.
Sobrini et al. (2021) [29]	40	Longitudinal	To analyse the malnutrition detection validity of the MNA-SF screening tool compared with GLIM criteria.	Patients older than 70 years with a recent diagnosis of cancer and a score <14 on the G8 screening tool.	6 months	MNA-SF is an effective tool to detect malnutrition in cancer patients with frail functional capacity, with 100% sensitivity and 50% specificity. Due to its high sensitivity, it can detect most cancer patients at nutritional risk. However, there is a disparity in the prevalence of malnutrition detected by the MNA, with 80%, compared with the GLIM criteria, which was 57.5%.
De Groot et al. (2020) [30]	246	Prospective cohorts	To assess the prevalence of malnutrition in outpatients and the validity of the PG-SGA tool and its reduced version compared with GLIM criteria.	Patients with gastrointestinal, lung, and head and neck cancers.	2 weeks	PG-SGA and PG-SGA SF were neither sufficiently sensitive nor specific to detect malnutrition and showed low concordance with GLIM.
Carretero & Ravasco (2014) [31]	460	Cross-sectional	To validate the MUST screening tool.	Cancer patients on outpatient chemotherapy.		MUST is valid for detecting malnutrition in oncology outpatients and shows good concordance with PG-SGA. The importance of the weight loss parameter in the MUST tool stands out, with a sensitivity of 49% and a specificity of 79%.
Tobberup & Thoresen (2020) [32]	120	Retrospective cross-sectional	To evaluate the accuracy of different screening tools in detecting malnutrition with reference to GLIM criteria.	Patients with unresectable lung cancer, using NRS-2002, MUST, SNAQ, PG-SGA SF, MST, MNA, and GLIM.		Concordance between NRS-2002 and MUST, with a higher specificity in MST and Nutriscore compared with NRS-2002 and MUST. While MNA-SF shows a higher rate of positive predictive value, its specificity is inadequate and its negative predictive value is low. In outpatients with lung cancer, PG-SGA stands out as the most effective tool to detect malnutrition.
Anzévui et al. (2012) [33]	95	Cross-sectional	To analyse the MSTC tool in outpatients.	Outpatients with any type of tumour.		MSTC screening tool is characterised as fast (5 min), practical and consistent. It has limitations in factors such as oedema, amputation, voluntary weight loss, cortisone use and extreme BMI.
Kuzma et al. (2014) [34]	76	Cross-sectional	To assess the need for nutritional screening in patients in the outpatient setting.	Breast, gynaecological, lung, and gastrointestinal.		In total, 52.6% of patients had MST 0, indicating no risk. Prevalence of malnutrition of 3.9% in outpatient centres.
